# Use of Deoxycorticosterone Pivalate by Veterinarians: A Western European Survey

**DOI:** 10.3390/vetsci8110271

**Published:** 2021-11-09

**Authors:** Rita Rebocho, Marina Domínguez-Ruiz, Ryane E. Englar, Carolina Arenas, Maria Dolores Pérez-Alenza, Andrea Corsini, Federico Fracassi, Michael Bennaim, Rodolfo Oliveira Leal

**Affiliations:** 1 Hospital Escolar Veterinário, Faculdade de Medicina Veterinária, Universidade de Lisboa, 1300-477 Lisboa, Portugal; ritacrebocho@hotmail.com; 2 Hospital Clinico Veterinario, University of Alfonso X el Sabio, 28691 Madrid, Spain; mdominguezvet@gmail.com; 3 College of Veterinary Medicine, University of Arizona, Oro Valley, AZ 85737, USA; renglar@arizona.edu; 4 Anicura Hospital Veterinario Valencia Sur, 46460 Silla, Spain; caroarenas10@hotmail.com; 5 Hospital Clinico Veterinario, Complutense University, 28040 Madrid, Spain; mdpa@vet.ucm.es; 6 Department of Veterinary Medical Sciences, University of Bologna, 40126 Bologna, Italy; andreacorsini.dvm@gmail.com (A.C.); federico.fracassi@unibo.it (F.F.); 7 Clinique Vétérinaire Aquivet, 33320 Eysines, France; michaelbennaim@gmail.com; 8 Centro de Investigação Interdisciplinar em Sanidade Animal, Faculdade de Medicina Veterinária, Universidade de Lisboa, 1300-477 Lisboa, Portugal

**Keywords:** DOCP, hypoadrenocorticism, deoxycorticosterone pivalate, fludrocortisone, Europe

## Abstract

This study aims to gather knowledge about the use of deoxycorticosterone pivalate (DOCP) by Western European Veterinarians (WEV) in dogs with typical hypoadrenocorticism. An observational cross-sectional study was conducted using an online survey, translated into four languages and disseminated to veterinary affiliates and mailing lists in six countries of Western Continental Europe. Respondents were tasked to share their therapeutic approach to hypoadrenocorticism, whether they preferred DOCP or fludrocortisone and the specific practical use of DOCP. One-hundred and eighty-four responses were included. Of these, 79.9% indicated that they preferred prescribing DOCP over fludrocortisone as a first-line treatment for mineralocorticoid supplementation. A total of 154 respondents had used DOCP at least once. Eighty percent of those who reported their initial dosage prescribed 2.2 mg/kg. After starting DOCP, 68.2% of the respondents assess electrolytes 10 and 25 days after administration following manufacturer instructions. In stable dogs, electrolytes are monitored quarterly, monthly, semi-annually, and annually by 44.2%, 34.4%, 16.9%, and 4.6% of respondents respectively. When treatment adjustment is required, 53% prefer to reduce dosage while 47% increase the interval between doses. Overall, DOCP is the preferred mineralocorticoid supplementation among WEV. Reported variability underlies the need to investigate the best strategies for DOCP use and therapeutic adjustments.

## 1. Introduction

Hypoadrenocorticism (hypoAC) is not commonly reported in companion animal practice, with an incidence of 2.3 cases per 10,000 dogs [1]. Medical management of primary typical hypoAC is lifelong and requires either treatment with deoxycorticosterone pivalate (DOCP), a long-acting synthetic mineralocorticoid ester with no effect on glucocorticoid activity or with fludrocortisone, a short-acting synthetic corticosteroid with potent mineralocorticoid activity and less glucocorticoid activity [2,3,4]. Because DOCP has no effect on glucocorticoid activity, it should be administered in conjunction with glucocorticoids in dogs with hypoAC.

The combination of subcutaneous injectable DOCP dosed approximately every 25–30 days and daily glucocorticoid supplementation is prescribed by many clinicians to manage hypoAC in dogs. This combination rapidly restores the sodium:potassium ratio, resolves clinical signs, and is easier to dose-adjust for mineralocorticoid replacement due to the absence of glucocorticoid activity [4,5,6,7,8,9].

Prior to DOCP approval by the United States Food & Drug Administration (FDA) in 1998 and by the European Medicines Agency in 2015, the standard mineralocorticoid supplementation for canine hypoadrenocorticism was fludrocortisone [10]. In 2015, a new formulation of DOCP was approved by the European Medicines Agency [11]. This formulation was subsequently commercialized in the United States in 2016. Although both formulations are licensed for veterinary use and are equally efficacious [12], only the new formulation of DOCP is available in Europe. Fludrocortisone must be prescribed off-label to treat dogs with hypoAC because there is currently no veterinary formulation.

The manufacturer of DOCP recommends that 2.2 mg/kg of DOCP be administered subcutaneously every 25 days with electrolyte monitoring at days 10 and 25 after the first dose to guide subsequent therapy [11]. Some veterinarians evaluate electrolytes for the first two months after the initial dose and following each dose adjustment to ensure that adequate mineralocorticoid support is provided [13]. After the patient has been stabilized with an appropriate dose and frequency of administration, electrolytes should be re-evaluated every 3 to 6 months [11]. Recently, some studies have suggested lowering the initial starting dose of DOCP [9,14,15] or reducing the frequency of administration [16]. These revised protocols effectively manage electrolytes and were developed out of concern by some veterinarians that standardprotocols might lead to biochemical evidence of overtreatment [15]. However, it is unknown which protocols are preferred by veterinarians in general practice.

This study aims to gather knowledge about the administration of DOCP by Western European Veterinarians (WEV) in dogs with typical hypoadrenocorticism.

## 2. Materials and Methods

An observational cross-sectional study was designed to gather information about treatment practices by surveying veterinarians in four languages (Portuguese, Spanish, French, and Italian) to solicit responses from Portugal, Spain, Italy, France, Belgium, and Luxembourg. Veterinarians were recruited to participate through a link to an online survey that was shared among forums specific to general practitioners as well as mailing lists.

The survey instrument consisted of 16 mandatory answers, multiple-choice or check-list style questions that focused on preferred initial treatment regimen, DOCP dosage, and therapeutic monitoring schedule in dogs that had been diagnosed with and were actively being treated for hypoAC. At the start of the survey, respondents were asked whether they had diagnosed at least one case of canine typical hypoAC within the previous 12 months. Only those respondents who answered in the affirmative (“yes”) could proceed with survey completion. The survey was divided into two sections. The first section focused on preferences between DOCP and fludrocortisone while the second section evaluated the protocol for the use of DOCP. In the first section of the survey, participants were asked about their preferences concerning whether they preferred fludrocortisone exclusively, fludrocortisone with glucocorticoid, DOCP exclusively, or DOCP with glucocorticoid. After disclosing their preferred treatment for hypoAC and their rationale, participants were asked if they had ever used DOCP in their clinical practice. Those who shared that they had never used DOCP were excluded from the study. Those who affirmed that they had in fact used DOCP in the past proceeded to the second section of the survey. This section solicited specific details about respondents’ protocols for using DOCP. Specifically, respondents were asked to specify their initial dose, when they recheck electrolytes after the first administration of DOCP, and how they adjust either DOCP dose or frequency. A copy of the survey is available in the Supplementary Materials.

Prior to dissemination, the survey was reviewed by six veterinarians and one epidemiologist. Surveys were accessible from 1 November 2019 through 31 May 2020. Participation was voluntary and no incentive was offered. No identifiers were attached to the survey; thus, all responses were anonymous. To be included in the study, participants were required to certify that they consented to the authors’ use of information.

The descriptive analysis required the use of spreadsheets through Microsoft Office Excel 2019^®^. For statistical analysis, the IBM SPSS Statistics^®^ tool was used.

## 3. Results

**Figure 1:**
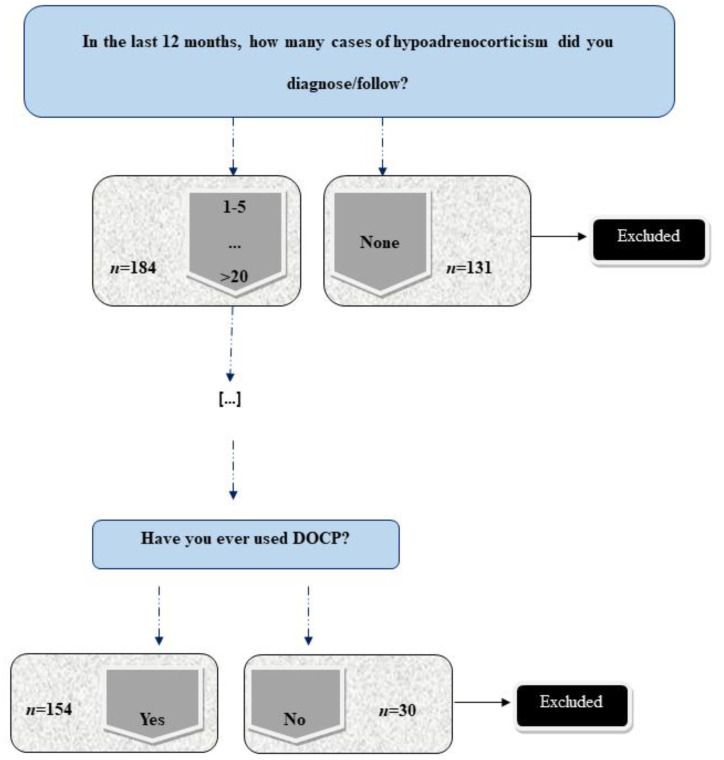
Survey’s exclusion criteria based on diagnosis of hypoadrenocorticism in the last 12 months and the use of deoxycorticosterone pivalate (DOCP). (“*n*” refers to number of responses).

Three-hundred and fifteen respondents provided data from a total of six different countries (Spain = 119, Portugal = 113, Italy = 38, France = 38, Belgium = 6, and Luxembourg = 1). One-hundred and thirty-one respondents (41.6%) were excluded because they had not diagnosed hypoAC within twelve months prior to data collection. Of the remaining 184, 30 respondents had never prescribed DOCP (Figure 1). This became a secondary exclusion criterion.

Considering the case load over the last 12 months, 95.7% (176/184) of respondents diagnosed 1 to 5 cases of hypoAC, 3.3% (6/184) diagnosed 5 to 10 cases, and 1% (2/184) diagnosed more than 10 cases.

### 3.1. Preference between DOCP and Fludrocortisone

A total of 79.9% of respondents (147/184) disclosed their preference for DOCP over fludrocortisone as their first-line approach for chronic management of hypoAC. Although 59.8% (110/184) reported combining DOCP with a glucocorticoid, approximately 20% of respondents (37/184) administer DOCP alone. Less than 10% of respondents (14/184; 7.6%) reported that they prescribe fludrocortisone solely (Table 1).

Those who preferentially chose DOCP over fludrocortisone (79.9%; 147/184) were asked to explain why. The most common rationales were that DOCP resulted in better clinical responses (60.5%; 89/147) and more effective long-term control of electrolytes (46.9%; 69/147) and that the product is licensed for use in veterinary medicine (45.6%; 67/147).

Those who preferentially prescribed fludrocortisone either alone or in combination with glucocorticoid (20.1%; 37/184) shared the following reasons: some were unfamiliar with DOCP (35.1%; 13/37), others had received positive feedback from clients of patients that had been prescribed fludrocortisone or the clinicians themselves had experienced prior positive patient outcomes with fludrocortisone (32.4%; 12/37). Other explanations for prescribing fludrocortisone preferentially included cost associated with DOCP (29.7%; 11/37) and the dog owner’s preference for a product that can be administered orally (21.6%; 8/37). Concerning dogs that were already receiving mineralocorticoid supplementation, and regardless of the initial therapy prescribed, 44% (81/184) of WEV reported that they have changed from fludrocortisone to DOCP at least once when treating a canine patient for hypoAC. In considering this transition, 39.5% (32/81) administer DOCP and progressively decrease the fludrocortisone dosage over 5 to 7 days, 30.9% (25/81) stop fludrocortisone first and administer DOCP on the following days, and 29.6% (24/81) administer DOCP and stop fludrocortisone on the same day. The main reasons for this transition were: a better clinical control (55.6%; 45/81) as evidenced by electrolyte stability (40.7%; 33/81), improved compliance (34.6%; 28/81) and the use of a licensed product for veterinary medicine (27.2%; 22/81).

A total of 56% (103/184) reported that they have preferentially maintained dogs on fludrocortisone even though DOCP is available for veterinary use because of positive patient response to fludrocortisone treatment (31.1%; 32/103), the higher cost associated with DOCP administration (24.3%; 25/103), owner’s preference for oral administration (21.4%; 22/103), familiarity with fludrocortisone (19.4%; 20/103), or availability issues of DOCP when it is not stocked at their workplace (19.4%; 20/103).

### 3.2. Protocol for the Use of DOCP

Of the 184 respondents included in the study, 30/184 (16.3%) reported never having prescribed DOCP in clinical practice. Their responses were excluded from this part of the study (Figure 1). Only answers from respondents who had prescribed DOCP at least once (154 respondents) were considered for review.

A total of 77.9% (120/154) disclosed their initial dosing protocol. Eighty percent (97/120) of the WEV administer an initial dose of 2.2 mg/kg while 10% (12/120), 6.7% (8/120), 1.7% (2/120), and 0.8% (1/120) use 1.5 mg/kg, 2 mg/kg, 1 mg/kg, and less than 1 mg/kg, respectively.

Administration of DOCP is performed by the veterinarian in 93.5% (144/154) of cases. In other cases, the administration is performed by veterinary nurses or technicians (3.3%; 5/154) or by owners who have been instructed to administer this medication at home (3.3%; 5/154).

Approximately two-thirds (68.2%; 105/154) of respondents measure electrolytes 10 and 25 days after initial DOCP administration, following the manufacturer’s instructions. Thirty-four (34/154; 22.1%) WEV monitor electrolytes on day 10 and day 28–30. Less than ten percent of WEV assess electrolytes once a month (9.7%; 15/154), citing cost as the primary deterrent against measuring electrolytes twice. Just over 5% (8/154), 2% (4/154) and 13% (3/154) of the respondents monitor electrolytes at day 10, 25 and day 28–30 respectively.

Concerning subsequent administrations of DOCP, 34.4% (53/154) of the respondents reassess patients every 25 days. However, 55.2% (85/154) assess electrolytes between days 28 and 30 (32.5% every 30 days and 22.7% every 28 days), citing the ease of scheduling with clients if the time interval between administrations is prolonged. About 10.4% (16/154) of the participants reported that they re-administer DOCP only in cases that involve clinical relapse.

Concerning adjustments in dosing, 72.7% (112/154) of WEV decrease the dose over time, according to the manufacturer’s recommendations. Eighteen out of 154 (11.7%) WEV maintain a dose of 2.2 mg/kg without adjustment, while 11% (17/154) start with a lower dose and progressively reduce it as needed.

A total of 64.9% (100/154) WEV shared what they prioritize when therapeutic adjustments are indicated. About 53% (53/100) prefer to reduce the dose first while 47% (47/100) prefer to start by increasing the dosing interval between administrations.

Regarding the long-term monitoring (Table 2), 44.2% (68/154) of participants reported checking electrolytes every three months while 34.4% (53/154) prefer checking every month. A smaller percentage of respondents (16.9%; 26/154) check electrolytes twice per year while 4.6% (7/154) assess them annually.

## 4. Discussion

This survey-based study is the first of its kind to detail DOCP use among WEV, confirming that it is the preferred drug used for mineralocorticoid supplementation in dogs with hypoAC.

The survey focused on two main points: the preference between products for mineralocorticoid supplementation and the practical use of DOCP. Despite the number of veterinary practitioners who completed the survey, a significant percentage did not meet the inclusion criteria of having diagnosed a case of hypoAC over the last year. Although more than half of the initial sample had previously diagnosed at least one case of hypoAC within the twelve months prior to survey completion, the great majority of the respondents reported diagnosing less than 5 cases, reinforcing that hypoAC is an uncommon disease in small animal practice. This is consistent with the literature and supports the suggestion that hypoAC is an uncommon endocrinopathy in companion animal practice [17].

The first segment of the survey tasked clinicians to share their preference between DOCP and fludrocortisone. As expected, a combination of DOCP and glucocorticoid is the most commonly prescribed therapy among WEV. Nonetheless, 20% of WEV reported administering DOCP as single-drug therapy, which can cause a lack of stabilization in the longterm [7]. Deoxycorticosterone pivalate has exclusive mineralocorticoid properties, lacking glucocorticoid activity. According to the author’s knowledge, only one case of isolated hypoaldosteronism is reported, supporting that single mineralocorticoid deficiency is an extremely rare condition in dogs [18]. Thus, DOCP should be systematically associated with glucocorticoid supplementation meaning that single-agent therapy with DOCP can be life-threatening for dogs with typical hypoAC [3,7]. These results reinforce the need for continuing education programs to clarify the proper use of prescribed agents, including DOCP, among general practitioners.

Some respondents still prefer to administer fludrocortisone to manage of canine hypoAC even though it must be prescribed in veterinary medicine as an off-label drug. A small percentage of WEV also reported using fludrocortisone without additional glucocorticoid support. Although some dogs may only require fludrocortisone, many require both for effective management and support [6]. In this select population of dogs, administering only fludrocortisone may potentiate challenges with patient stabilization, even though fludrocortisone use alone can rapidly improve clinical signs, such as appetite. Owners may report satisfaction with therapy on account of improving clinical signs even if patients may or may not be well regulated from the clinician’s perspective [6].

Improved patient response and clinical outcome are the primary reasons why some WEV preferentially prescribed DOCP over fludrocortisone. This finding aligns with Baumstark et al. (2014), who verified a faster normalization of electrolytes and clinical signs with DOCP. In addition, the same authors showed that renin activity, a reliable tool for monitoring mineralocorticoid treatment, decreases in dogs treated with DOCP rather than with fludrocortisone. Therefore, there is evidence supporting the use of DOCP such that it might be more frequently prescribed among practitioners over fludrocortisone in dogs that require concurrent mineralocorticoid therapy [5]. Apart from the already documented benefits, DOCP is also preferred because it is licensed for veterinary use, meaning it should be considered the first choice for use in dogs according to the EU’s law. Sieber-Ruckstuhl et al. (2019) observed that DOCP, being an injectable suspension, can be easier than fludrocortisone when it comes to compliance, particularly for those owners who cannot administer fludrocortisone per os. Results from this study agree with the literature as the owner’s compliance was an evoked reason for the preferable use of DOCP. Despite the evoked arguments, a minor percentage of the participants still prefer using fludrocortisone, justifying they are not familiar with DOCP or have had positive patient outcomes or client feedback with fludrocortisone. Lack of familiarity with DOCP may be because it was only recently approved by the European Medicines Agency^®^. Although there is enough information about its correct use, fludrocortisone was considered to be the gold standard for mineralocorticoid supplementation over many years. Despite some variabilities among countries, fludrocortisone also tends to be more affordable than DOCP, making some dog-owners more likely to treat canine hypoAC [19]. Oral administration is also likely to be more practical and easier to manage outside of the clinic.

Concerning dogs with hypoAC that already receive fludrocortisone therapy, most respondents do not switch them over to DOCP because the dogs have responded well to fludrocortisone. Respondents also expressed concern that there may be more risk associated with changing the protocol than maintaining the current therapy on which the dogs are already stable. Due to its rapid effect, it might not be necessary to overlap fludrocortisone administration with DOCP injection [20].

The second segment of the survey focused on protocols for DOCP use. More than 60% of the WEV follow the manufacturer’s instructions, initiating treatment at 2.2 mg/kg. The authors suspect that this indicates a reluctance to employ lower doses, although several studies have already demonstrated that lower doses are efficacious and clinically safe [9,14,15,21]. In fact, those that use lower doses reported positive patient outcomes and fewer side effects, as reported in previous studies, highlighting that in several dogs, a lower dose can be safely considered. The exception is younger dogs, in which starting with lower dosages might not be sufficient, and a higher dose than 1.5 mg/kg is advisable [9]. Nonetheless, due to the unpredictable individual response to treatment, this is still a point of discussion and for a safer practice, the recommended dosage is preferred by the respondents.

The participants stated that the administration of DOCP is commonly performed by veterinarians even though owners can be taught to administer subcutaneous in the same way that they might administer insulin injections. There is room for improvement when it comes to client education. This strategy has the potential to decrease costs and minimize the stress of the dog, despite the potential legal concerns.

Manufacturer instructions and the current veterinary medical literature advise that clinicians should evaluate electrolytes at day 10 following the first DOCP injection for dose adjustment and again at day 25 to adapt frequency [13]. Although the majority of the WEV comply with these recommendations, approximately 10% of the participants only assess electrolytes onceamonth to reduce costs. This finding is concerning because routine monitoring of electrolytes is essential to maximize therapeutic success. When cost is an issue, rather than minimizing electrolyte controls, other options can be considered such as decreasing DOCP dosage or frequency of administration. However, decreasing frequencyor decreasing both dose and frequency might increase the likelihood of decompensation of the disease [16]. In this study, about one-half of the respondents prefer decreasing the frequency of administration to 28–30 days due to compliance and to facilitate the administration schedule. This can be supported by a previous study [16], showing that the DOCP effect can last more than the 25-day recommended period. The other half of respondents prioritize a decrease in dosage instead of frequency. These results highlight the current variability in DOCP dose andr dosing interval among WEV.

It is noteworthy that a minority of participants administer DOCP only when dogs start to be clinically destabilized. This increases the risk of an Addisonian crisis and stresses the need for continuing education about the potentially life-threatening consequences of a poorly managed chronic hypoAC.

Approximately 80% of respondents engage in long-term electrolyte monitoring, checking electrolytes once per month or, at minimum, once every three months. There is an ongoing debate about whether a monthly approach is overzealous or whether these check-ins are essential in a stable patient.

A primary limitation for this study is that some participants were not congruent in related answers, making it challenging for the authors to discern which answer was most accurate. For instance, two respondents stated that DOCP is the preferred treatment but reported that they had never used it before. This discrepancy may eventually be explained by external reasons (such as availability of the product or costs) and is out of the scope of this project. Second, the survey did not focus on questions about the side effects of DOCP. It would have been helpful to clarify how often the adverse effects take place in general practice. Finally, DOCP dosage was questioned in a closed-ended manner meaning that intermediate doses (such as 1.2 mg/kg or 1.8 mg/kg) were not included among the options.

## 5. Conclusions

This study supports that DOCP is the preferred treatment for mineralocorticoid supplementation of dogs with hypoAC in WEV. However, no clear consensus exists among WEV if it is the dose or the dosing interval that would require adjusting among dogs under treatment. Reported variability underlies the need to investigate the best strategies for DOCP use and therapeutic adjustments.

## Figures and Tables

**Table 1:** First-line treatment preference for hypoAC in dogs among WEV.

Therapeutical Options	[*n* (%)] *n* = 184
DOCP + Glucocorticoid	110 (59.8%)
Exclusively DOCP	37 (20.1%)
Fludrocortisone + Glucocorticoid	23 (12.5%)
Exclusively Fludrocortisone	14 (7.6%)

**Table 2:** Long-term monitoring frequency of dogs treated for hypoAC among WEV.

Frequency	[*n* (%)] *n* = 154
Once a month	53 (34.4%)
Every 3 months	68 (44.2%)
Every 6 months	26 (16.9%)
Annually	7 (4.6%)

## Data Availability

The data presented in this study are available on request from the corresponding author.

## Supplementary Materials

The following are available online at https://www.mdpi.com/article/10.3390/vetsci8110271/s1, —Copy of the survey—“Use of Deoxycorticosterone Pivalate by Veterinarians: a Western European Survey”.
