# Supplementary material for: Use of Deoxycorticosterone Pivalate by Veterinarians: A Western European Survey
Source: Vet Sci. 2021 Nov 9;8(11):271. doi: 10.3390/vetsci8110271 (PMC8624386; doi:10.3390/vetsci8110271)
Supplement: Supplementary file 1 [file vetsci-08-00271-s001.zip › vetsci-1413580-supplementary.pdf]

Supplementary file: Use of Deoxycorticosterone Pivalate by Veterinarians: a Western European Survey

This survey is anonymous and data are confidential. Please, be honest in your answers, so we can get valid and representative conclusions. When filling it, the respondent certifies that he/she consents to the author's use information and agrees that data can be used for scientific study purpose and further publication.

1. In the last 12 months, how many cases of hypoadrenocorticism did you diagnose/follow?

- I did not diagnose/follow any case over the last 12 months
- 1-5
- 5-10
- 10-20
- >20

Section 1 - *Preference between DOCP and Fludrocortisone*

2. In animals recently diagnosed with hypoadrenocorticism, which one of the following drugs do you usually use for treatment?

- Fludrocortisone
- Deoxycorticosterone Pivalate
- Prednisone/Prednisolone + Fludrocortisone
- Prednisone/Prednisolone + Deoxycorticosterone Pivalate

3. If you used DOCP why did you use it? (You can tick more than 1 option)

- Because DOCP is licensed for veterinary use
- Due to owner's *Compliance*
- There is a better electrolytic control and for a long term with DOCP
- Because it is a new compound
- Because, after treatment with Prednisone/Prednisolone + Fludrocortisone, the animal had some clinical side effects due to excess of glucocorticoids
- Better clinical control of hypoadrenocorticism with DOCP

4. If you used fludrocortisone, why did you use it? (You can tick more than 1 option)

- I do not know DOCP
- I know DOCP, but I don't feel comfortable using it
- I have experienced hypersensitivity to DOCP in previous cases
- I'm afraid about drug interactions with DOCP
- I have dogs with other concomitant diseases (e.g. congestive heart disease, renal disease, hepatic disease…) that can compromise the use of DOCP
- Pregnant bitches
- Lower cost comparing to DOCP
- I have good feedback and experience with Fludrocortisone
- The owners prefer an oral administration
- I don't have DOCP in stock

5. Have you ever switched Fludrocortisone to DOCP in a dog with typical HypoAC already on treatment?

- Yes
- No

6. If you answered no (on question 5), why did you not switch for DOCP? (You can tick more than 1 option)

- I have a good positive response to Fludrocortisone
- I am more familiarized with Fludrocortisone
- DOCP is more expensive
- Because treatment with Fludrocortisone works well
- I do not know DOCP
- I know DOCP, but I don't feel comfortable using it
- I have experienced hypersensitivity to DOCP in previous cases

- I'm afraid about drug interactions with DOCP
- I have dogs with other concomitant diseases (e.g. congestive heart disease, renal disease, hepatic disease…) that can compromise the use of DOCP
- Pregnant bitches
- Lower cost comparing to DOCP
- I have good feedback and experience with Fludrocortisone
- The owners prefer an oral administration
- I don't have DOCP in stock

7. If you answered yes (on question 5) - Why did you switch for DOCP? (You can tick more than 1 option)

- Because DOCP is licensed for veterinary use
- Due to owner's *Compliance*
- There is a better electrolytic control and for a long term with DOCP
- Because it is a new compound
- Because, after treatment with Prednisone/Prednisolone + Fludrocortisone, the animal had some clinical side effects due to excess of glucocorticoids
- Better clinical control of hypoadrenocorticism with DOCP

8. If you answered yes (on question 5) - How did you perform the transition?

- I've administered DOCP and stopped Fludrocortisone on the same day
- I've administered DOCP and progressively reduced the Fludrocortisone dosage during 5 - 7 days
- I stopped Fludrocortisone and in the following day I started DOCP

9. Have you ever used DOCP?

- Yes
- No

Section 2 - *Protocol for use of DOCP*

10. If you use DOCP, which initial dose do you recommend?

- 2.2 mg/kg
- 2 mg/kg
- 1.5 mg/kg
- 1 mg/kg
- < 1 mg/kg
- Do not remember

11. Who is the person responsible for DOCP administration?

- Veterinarian
- Owner (after instructions)
- Veterinary Nurse/Tech

12. After the first DOCP dose, when do you monitor electrolytes?

- At day 10 and day 25 after injection
- At day 10 and day 28-30 after injection
- Only at day 10 after injection
- Only at day 25 after injection
- Only at day 28-30 after injection

13. After the first dose of DOCP, when do you evaluate/reassess dogs for a subsequent administration?

- Every 28 days (ex: Monday of every month)
- Every 30 days (ex: day 1 of every month)
- Every 25 days (as indicated in the package insert)
- When dogs start deteriorating

14. Based on your experience, when a dog under DOCP is clinically stable, do you change the current dose?

- I tend to reduce the dose following manufacturer's recommendations
- Usually, I don't reduce dose and keep the initial one (2.2mg/kg)
- I just start the treatment with a lower dose, so I don't reduce it
- I start the treatment with a lower dose and thereafter, I still reduce it progressively

15. Concerning therapeutic adjustments in a medium-term perspective, what do you prioritize in a stable dog? Do you increase the time between DOCP administrations or do you decrease its dose?

- First, I tend to reduce the dose before extending the period between injections
- First, I increase the period between injections before reducing the dosage
- I follow manufacturer’s recommendations in order to avoid worsening of clinical signs in a medium-term

16. In a long term perspective, when do you monitor dogs under DOCP treatment?

- Every month
- Every 3 months
- Every 6 months
- Once a year
